# Violacein-Producing *Collimonas* sp. from the Sea Surface Microlayer of Costal Waters in Trøndelag, Norway

**DOI:** 10.3390/md7040576

**Published:** 2009-11-12

**Authors:** Sigrid Hakvåg, Espen Fjærvik, Geir Klinkenberg, Sven Even F. Borgos, Kjell D. Josefsen, Trond E. Ellingsen, Sergey B. Zotchev

**Affiliations:** 1 Department of Biotechnology, Norwegian University of Science and Technology, N-7491 Trondheim, Norway; E-Mails: sigrid.hakvag@biotech.ntnu.no (S.H.); espen.fjarvik@biotech.ntnu.no (E.F.); sven.borgos@biotech.ntnu.no (S.E.F.B.); 2 SINTEF Industrial Biotechnology, SINTEF, N-7034 Trondheim, Norway; E-Mails: geir.klinkenberg@sintef.no (G.K.); kjell.d.josefsen@sintef.no (K.D.J.); trond.e.ellingsen@sintef.no (T.E.E.)

**Keywords:** surface microlayer, *Collimonas*, violacein, biosynthetic genes

## Abstract

A new strain belonging to the genus *Collimonas* was isolated from the sea surface microlayer off the coast of Trøndelag, Norway. The bacterium, designated *Collimonas* CT, produced an antibacterial compound active against *Micrococcus luteus*. Subsequent studies using LC-MS identified this antibacterial compound as violacein, known to be produced by several marine-derived bacteria. Fragments of the violacein biosynthesis genes *vioA* and *vioB* were amplified by PCR from the *Collimonas* CT genome and sequenced. Phylogenetic analysis of these sequences demonstrated close relatedness of the *Collimonas* CT violacein biosynthetic gene cluster to those in *Janthinobacterium lividum* and *Duganella* sp., suggesting relatively recent horizontal gene transfer. Considering diverse biological activities of violacein, *Collimonas* CT shall be further studied as a potential producer of this compound.

## 1. Introduction

The genus *Collimonas* was described for the first time in 2004 [1]. These bacteria were isolated from slightly acidic dune soils in the Netherlands, and are strictly aerobic, Gram-negative rods. *Collimonas fungivorans gen. nov., sp. nov*., are chitinolytic and able to grow on living hyphae of several soil fungi. Based on 16S rDNA sequences, the most closely related genera are *Herbaspirillum* and *Janthinobacterium.*

Violacein (3-[1,2-dihydro-5-(5-hydroxy-1*H*-indol-3-yl)-2-oxo-3*H*-pyrrol-3-ylidene]-1,3-dihydro-2*H*-indol-2-one) is a blue-black indole-derived pigment described already in 1882. The violacein carbon skeleton is produced from two molecules of L-tryptophan, and molecular oxygen is required for production of the pigment [2–4]. The role of violacein production in the bacteria is not understood, but it has been suggested that it gives a survival advantage in the competition with other microorganisms in the environment [5]. Other suggestions include involvement in protection against visible radiation and regulation of tryptophan production, which is toxic for bacteria at high concentrations [6 and reference therein]. Violacein is produced by several bacterial species, including the Gram-negative species *Chromobacterium violaceum*, *Janthinobacterium lividum*, *Pseudoalteromonas luteoviolacea*, *Ps. sp* 520P1 and *Ps. sp.* 710P1 [7–9]. Recently the violacein biosynthetic gene cluster from the Gram-negative *Duganella* sp. B2 was submitted to GenBank [10]. These strains have been isolated from water and soil in tropical and subtropical regions, rivers, lakes and springs and from seawater at a depth of 320 m outside Japan.

**Figure 1.**
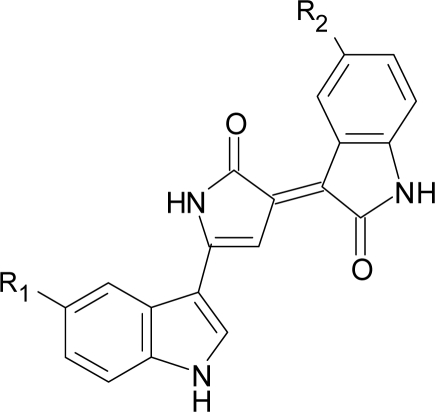
Chemical structures of violacein (A) and deoxyviolacein (B). **A**: Violacein: R_1_ = OH, R_2_ = H **B:** Deoxyviolacein: R_1_ = R_2_ = H

The gene cluster for violacein biosynthesis has been sequenced from several of the violacein producers, including *Ch. violaceum* and environmental DNA [11,12]. The 8 kb and 6.7 kb violacein clusters have been reported to contain four genes (*vio*A-D) responsible for the production of violacein and deoxyviolacein [4] (Figure 1). A fifth gene (*vio*E) has later been described as being essential for violacein biosynthesis [13].

Violacein has shown anti-protozoan [14,15], anticancer [16,17], anti-viral [18], antibacterial (both G+ and G−) [13,19,20] and antioxidant [21] activities. The antibacterial activity includes inhibition of Staphylococcus aureus, Neisseria meningitidis, Streptococcus spp., Bacillus spp., Mycobacterium and *Pseudomonas,* among others.

Based on these properties, violacein would seem to be commercially interesting for therapeutic purposes and it has in fact been proposed for dermatological purposes [6]. It has been suggested that violacein should be considered an *in vitro* genotoxic compound to mammalian cells, (due to its toxicity in VERO and FRhK-4cells), but further investigations are needed before drawing any conclusions on violacein’s future pharmaceutical potential [22].

Up to now *Ch. violaceum*, which is the best studied violacein producer, has not been widely utilized for commercial purposes. One reason might be that it can act as an opportunistic pathogen in humans [23]. It would therefore be interesting to isolate new producers of violacein.

In this study, a new strain of the genus *Collimonas* has been isolated, and examined for its antimicrobial potential. The production of a characteristic blue pigment and the demonstrated antibacterial activity seem to be ascribed to violacein biosynthetic genes. These findings suggest that this bacterium might be interesting for the biotechnological industry and prompt further studies.

## 2. Results and Discussion

### 2.1. Isolation of Collimonas CT

In this study, four bacteria producing a blue pigment were isolated from the sea surface microlayer at the coast of Trøndelag, Norway. Sequencing of partial 16S rDNA sequences (1490 bases) from the four strains revealed two unique sequences that were 99.3% identical. Both displayed 98.8% identity to *Collimonas fungivorans* CTE227. The isolates are therefore named *Collimonas* CT (Coast of Trøndelag) in this article. Other *Collimonas sp*. has been isolated from terrestrial sources, mainly soil [1,24]. One extremophile *Collimonas sp.* has been isolated from submarine ikaite columns in Greenland [25] and another strain has been isolated from stream water in Finland [26]. Initial cultivation of the seawater samples from the coast of Trøndelag was performed on media containing nalidixic acid, to minimize growth of Gram-negative bacteria. Isolation of the Gram-negative *Collimonas* CT from these samples indicates that the bacteria are able to grow in the presence of this antibiotic at the concentrations used. Resistance to nalidixic acid has also been observed for *Janthinobacterium lividum* and *Chromobacterium violaceum* [27,28].

*C. fungivorans* strains show high sequence similarity to representatives of the genus *Janthinobacterium* (~95%) and *Herbaspirillum* (~96%), and are reported to display the highest growth rates at 20–30 ºC [1]. As for *Collimonas* CT, an increase in incubation temperature from the water temperature at the sampling site (ca 13 ºC) to 20 ºC and 25 ºC increased the growth rate, and did not inhibit pigment production. The *Collimonas* CT bacteria did not produce pigment when cultivated at 30 ºC and did not grow at 37 ºC. Loss of pigment production when incubated at 25 ºC or higher has also been observed by others [26].

To optimize the conditions for production of antimicrobial compounds, the isolates were cultivated on four different production media, with or without 50% seawater. Interestingly, the isolates grew slower or displayed no growth on media containing seawater. Some pigment production could be seen in the growing cultures, but probably due to the poor growth the antimicrobial activity was very low in the extracts of such cultures. The inhibited growth of the bacteria on media containing seawater indicates that they might be of terrestrial origin.

### 2.2. Identification and characterization of antimicrobial compound and pigment

Antimicrobial activity of *Collimonas* CT was assayed with *Micrococcus luteus* (ATCC 9341)*, Candida albicans* (ATCC 10231), *Escherichia coli* K12, *Enterococcus faecium* CCUG 37832 and *E. faecium* CTC 492 as indicator organisms. Activity could only be detected against *M. luteus* under the production and assay conditions tested (see experimental section). The antibacterial activity of violacein against *E. coli* is reported to be low, even at high concentrations [29,20].

Extracts from *Collimonas* CT showing antibacterial activity were fractionated by LC-fractionation and analyzed by LC-MS as described in Section 3.5. After the first fractionation step, a blue/purple color was observed in fractions 6 and 7 (eluting at 7 to 9 minutes from injection). The bioactivity of the fractions was measured against *M. luteus* in an agar diffusion assay (see Section 3.4), and the bioactivity was found in the same fractions as the colored compound. These fractions were pooled and fractionated again at two different conditions (see Section 3.5), and the bioactivity of the fractions was measured as described above. At both conditions, the colored fractions (fraction 5 collected 5 to 6 minutes after injection at condition I and fraction 6 collected 6 to 7 minutes at condition II, se Section 3.5) were bioactive against *M. luteus*. The bioactive fractions were analyzed by LC-MS TOF (data not shown) and the resulting molecular masses (10 ppm window) were submitted to the online version of the Dictionary of Natural Products (http://dnp.chemnetbase.com/). The database search indicated that the molecular mass that most likely corresponded to the dominant DAD-profile observed in the fractions was close to the reported mass for Violacein in the database (approximately 5 ppm off). A larger amount of this compound was therefore purified as described in last part of Section 3.5, and the following section presents results from analysis of the purified compound. The UV (DAD) absorbance plot (data mot shown) four peaks, of which the peaks at 12.5 and 15.5 minutes showed similar UV profiles. The main compound in the sample, eluting at 12.5 minutes, had an m/z = 342.0882, whereas the compound eluting at 15.5 minutes had an m/z value of 326.0938. The measured masses deviates 0.6283 and 0.9188 ppm from the stoichiometric formulas ([M-H]^−^ ion) of C_20_H_13_N_3_O_3_ and C_20_H_13_N_3_O_2_. Based on measurements at the UV absorbance maximum of 572 nm, the relative abundance of the latter compound was 49.6% of the former. Based on the MS-analysis, colour of the substrate and a UV-profile similar to that of violacein (http://dnp.chemnetbase.com/), the two main compounds were assumed to be violacein (C_20_H_13_N_3_O_3_) and deoxyviolacein (C_20_H_13_N_3_O_2_).

The compounds eluting at 9.5 minutes and 13 minutes had m/z values deviating less than 1.4 ppm from the molecular ion stochiometries of C_15_H_9_O_4_ and C_15_H_9_O_5_, respectively. Relative amounts of these two compounds could not be estimated from the UV data as their extinction coefficients are not known.

As previously mentioned, *Collimonas* CT isolates did not produce pigment when grown at 30 ºC. Temperature, agitation and pH also affect violacein production in *Ch. violaceum* [as cited by 30]. Colourless colonies were also at one point observed in re-streaks of the CT-isolates. This phenomenon is not unknown among violacein producers [30,31]. Sequencing of 16S rDNA, and PCR with degenerate primers for *vio*A- and *vio*B- biosynthesis genes confirmed that these isolates were in fact potential violacein producing *Collimonas sp*. Inhibition assays revealed that antimicrobial activity was lost in extracts from the white colonies, indicating that colour and antimicrobial activity are linked. The UV-profile of extracts from the colourless mutants confirmed that no violacein was present in the sample, and that the production of violacein was lost. These experiments show that violacein is not essential for growth as also reported earlier for other violacein producers [32].

Fractionation of the bacterial extracts followed by antimicrobial assays revealed that the antibacterial activity was found in the same fractions as the blue pigment, indicating that the activity found in *Collimonas* CT extracts is due to the blue pigment. The reported antibacterial activity of violacein is mostly against Gram-positive bacteria, which is in accordance with the observed activity against *M. luteus*. These results confirm that the observed antibacterial activity of the *Collimonas* CT isolates most probably is not caused by several compounds, and that the main bioactive compound is violacein.

**Figure 2.**
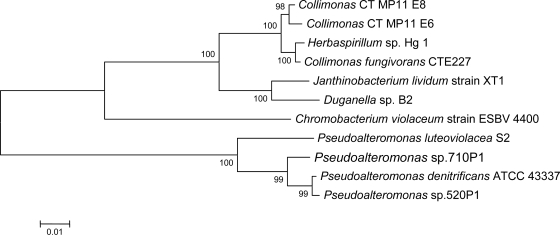
Phylogenetic relationship between partial 16S rDNA sequences (1354 bp) of known violacein producers, using the neighbor-joining method with 2000 bootstrap replicates. Closest matches from the BLAST search, *Collimonas fungivorans* CTE227, and *Herbaspirillum* sp. Hg1 (both not known to produce violacein) are also included in the tree. Two *Collimonas sp* from this study are displayed.

As earlier described, *Collimonas* CT 16S rDNA is highly similar to *Janthinobacterium* and *Herbaspirillum*. Production of violacein is a characteristic of *Janthinobacterium* [33]. Despite the close relationship, *C. fungivorans* has not been reported to produce violacein even though assumed violacein producing strains of *Collimonas* has been described [26]. Violacein non-producing strains of both *Janthinobacterium* and *Chromobacterium violaceum* have also been described [32,34]. The phylogenetic relationship between the partial 16S rDNA sequences from the aforementioned species is shown in Figure 2.

Despite that the *Collimonas* CT bacteria were isolated from marine samples, the isolates show inhibited (or no) growth on seawater-containing media. Violacein has earlier been found in bacteria isolated from marine environment, and this might suggest that the *Collimonas* CT are growing as biofilm in the tidal zone of brackish water, or in soils/fresh water and had been washed out into the sea not long before sampling.

### 2.3. Violacein biosynthesis genes in Collimonas CT

Initial screening for *vio*A and *vio*B sequences was performed to substantiate the assumption that the produced pigment was in fact violacein. *vio*A- and *vio*B-fragments were obtained by PCR with degenerate primers. It is reported that disruption of *vioA* or *vioB* would completely abrogate the biosynthesis of violacein [11]. Attempts to inactivate *vio*B by homologous recombination in this study gave rise to white colonies without antimicrobial activity. Results from the following Southern blotting with the *vio*B fragment as probe were inconclusive, probably due to large deletion in the mutants.

**Figure 3.**
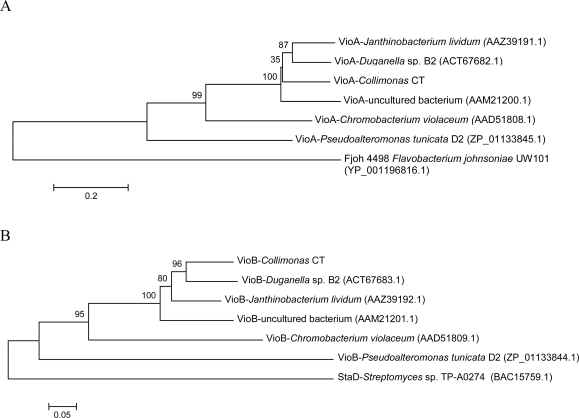
Phylogenetic trees constructed of the amino acid sequences of VioA (**A**) and VioB (**B**) proteins (313 and 309 amino acids) from known violacein producers. The analysis is performed using the neighbor-joining method with 1000 bootstrap replicates. Partial sequences for Fjoh_4496 and chromopyrrolic acid synthase, StaD are the best non-VioA/B hits from BLAST searches, and are included as out-roots. Accession numbers for the sequences are given in parentheses.

Phylogenetic analyses of amino acid sequences of partial *vioA* and *vioB* genes from different violacein producers were performed. The resulting trees, shown in Figure 3, shared similar topology, suggesting that the *vio*- genes in the different strains might share the same evolutionary history.

A comparison of the similarity of the VioA- and VioB protein fragment from different violacein producers to the proteins from *Collimonas* CT was performed. 16S rDNA of *Collimonas* shows higher degree of identity to *Janthinobacterium lividum* (formerly *Chromobacterium lividum* [33]), and *Duganella* sp. B2 (94%) than to *Chromobacterium violaceum* (88%), as shown in Figure 2. This is also the case with the VioA- and VioB-fragments from *Collimonas* CT (proteins from uncultured bacterium not included). In average the protein fragments displayed ~78% identity to VioA/B fragments from *J. lividum* and *Duganella* sp B2 and ~53% identity to VioA/B fragments from *Ch. violaceum.* These data suggest that the violacein biosynthetic gene cluster might have been subject to relatively recent horizontal gene transfer between *Collimonas*, *Janthinobacterium* and *Duganella* species.

## 3. Experimental Section

### 3.1. Sampling sites and sample collection

The *Collimonas* CT strains were isolated from the neuston layer at two locations along the coast of Trøndelag, Norway. Water samples were collected on the 18th of June 2004 in Snillfjord (63º 23,755 N, 009º 29,327) and the 1st of July 2004 at Sula (63º 50,595 N, 008º 27,552 E). The water temperatures were 12.4 and 13.8 °C, respectively, and the salinity corresponded to 18.5 and 33.1 practical salinity units (psu). The surface microlayer was collected using teflon plates and the bacteria isolated as earlier described [35]. Both samples were collected around high tide and 2 to 3 meters from the shoreline. Table 1 lists the microbial strains and plasmids used in this study.

The *Collimonas* CT strains were initially isolated on Kusters streptomycete isolation agar (2% w/v) (modified); Glycerol (10 g), Casein (0.3 g), KNO_3_ (2 g), FeSO_4_ ·7 H_2_O (0.25 mg), H_2_SO_4_ (0.5 mg), natural sea water (0.5 L) and distilled water (0.5 L), pH 8.2, supplied with Cycloheximide (50 μg/mL) and Nalidixic acid (30 μg/mL).

### 3.2. Preparation of bacterial inoculums

A mixture of 1–4 colonies with 5 g glass beads and 2.5 mL 0.9% NaCl with 0.1% Tween 20, was whirlmixed for one minute. Whirlmixing was repeated after 15 min. The cell-material was centrifuged and washed twice with sterile water, before resuspension in 1.5 mL sterile water.

### 3.3. Culture condition for production and extraction of secondary metabolites

The isolates were cultivated on different 1% agarose (production) media to facilitate production of secondary metabolites. Initial cultivation, extraction and antimicrobial assays were performed as described earlier [35]. A fourth production medium (PM1) was included, containing: Malt extract (5 g), yeast extract (2 g), glucose (2 g), agarose (10 g) and tap water (1 L), pH 8.2. PM1 is identical to ½ ISP2 medium used in the initial cultivation of the bacteria, except for the use of tap water, and exchanging the agar (2%) with agarose (1%). Cultivation on production media with 50% seawater was also performed. To identify the optimal incubation time for production of bioactive compounds, different incubation time points (1 to 9 days) and two incubation temperatures (20 and 30 ºC) were tested for each medium. Extraction was performed with both ethyl acetate and methanol in parallel with DMSO.

Upscaled tests, with reduced number of production parameters, were performed by cultivation in flat 6-well Tissue Culture Plates (Sarstedt nr 83.1839.500) with 1.5 mL agarose media in each well, inoculated with 25 μL inoculum. Extraction was performed with 2.5 mL DMSO. Dried cultures on agarose media was crushed, and incubated with DMSO and glass beads on a rotator for 2 hours in the dark.

Cell extracts for liquid assays were prepared from supernatant and pellet of liquid cultures. Initial tests based on results from cultivation on solid media and agar diffusion assays, were performed to determine the optimal incubation time and production medium. Precultures of *Collimonas* CT were prepared by inoculating colonies from plates, and growing them in PM2 (production medium 2) for 16 hours at 20 ºC. PM2 contained; Mannitol (20 g), soybean flour (20 g), Clerol (antifoam, 0.5 g), dry yeast (3.4 g), and tap water (1 L). Fresh medium (30 mL) was inoculated 3%, and incubated for three days at 20 ºC. The bacteria were pelleted by centrifugation (10,000 × g, 10 minutes). The pellet and the supernatant were freeze dried separately. Freeze dried material was extracted with equal volumes of DMSO.

### 3.4. Antimicrobial assay

The bacterial extracts were stored at −20 ºC, and tested in agar diffusion (a.d.) and liquid assays (l.a.) for antagonistic activity against *Micrococcus luteus* (ATCC 9341)*, Candida albicans* (ATCC 10231) and *Escherichia coli* K12 (a.d. only), and *Enterococcus faecium* CCUG 37832 and CTC 492 (l.a. only). Agar diffusion- and liquid assays were performed as described earlier [35,36]. Only DMSO extracts from bacteria grown on PM2 and PM3 were tested in the liquid assays.

### 3.5. Fractionation and LC-MS-TOF analysis of bacterial extracts

Samples of selected DMSO-extracts were fractionated using an Agilent 1100 series HPLC system equipped with a diode array detector (DAD) and a fraction collector. Each sample was fractionated using two different types of LC-columns: (1) Agilent ZORBAX Eclipse XDB-C18, 5 μm, 4.6 × 150 mm and (2) Agilent SB-CN 3.5 μm, 4.6 × 75 mm. For both types of columns, a flow of 1 mL/min of a mixture of 0.005% formic acid in deionized water and acetonitrile was used as mobile phase. In both cases the concentration of acetonitrile was kept at 25% the first minute, then increased linearly from 25 to 95% during the next 11 minutes and kept at a concentration of 95% for the rest of the run. The fraction collector was used to collect 12 fractions of the eluent from 1 minute until 13 minutes from injection. The samples were first fractionated using LC-column (1). The fractions displaying antibacterial activity (see below) were further fractionated in parallel using conditions (1) and (2).

The samples from LC-fractionation were dried in a vacuum centrifuge (Savant Speed-Vac), dissolved in DMSO and the bioactivity of the fractions determined in an agar diffusion assay using *M. luteus* as indicator organism [35]. Selected samples from the second LC-fractionation that showed bioactivity were further analysed using an Agilent 1100 series HPLC system connected to a diode array detector (DAD) and a time of flight (TOF) mass spectrometer. The LC conditions used were as described for condition (2) above. Electrospray ionization was performed in negative mode (350 ºC gas temperature, 12 L/min drying gas, 50 PSI nebulizer pressure). The DAD plots were used to identify the approximate retention times of the bioactive compounds in the fractionation runs and in the LC-MS-TOF analysis. Molecular masses corresponding to significant peaks identified in bioactive samples from parallel fractionations (C18 and CN columns) were compared and molecular masses common to fractions from the C18 and CN columns were identified.

Bacterial DMSO extracts were purified on a C18 solid-phase extraction column, 55–105 μm (Waters nr.WAT036945), and eluted with methanol. The methanol solutions of the purified compound were analyzed on an Agilent 1100 HPLC system equipped with a diode array detector (DAD) and an Agilent time-of-flight (TOF) mass spectrometer with an electrospray ion source run in negative mode (350 ºC gas temperature, 12 L/min drying gas, 50 PSI nebulizer pressure). For the LC separation, a Bonus-RP column (3.5 μm, 2.1 × 50 mm, Agilent Technologies, USA) was used. The mobile phase consisted of a mixture of 10 mM ammonium acetate (pH 4.0) and acetonitrile at a flow of 0.3 mL/min. The concentration of acetonitrile was kept at 25% the first two minutes, then increased linearly from 25 to 90 % during the next 24 minutes and kept at a concentration of 90% for the remaining 2 minutes.

### 3.6. Cloning, sequencing and phylogenetic analysis

Total-DNA of the bacteria was isolated using DNeasy Blood & Tissue Kit (Qiagen) according to manufacturer’s protocol. PCR with bacteria specific primers, BP_F27: 5′-AGA GTT TGA TCM TGG CTC AG-3′ and BP_R1492: 5′-TAC GGY TAC CTT GTT ACG ACT T-3′, was performed to amplify 1.5 kb of the 16S rRNA gene [37].

The PCR was performed using initial denaturation at 94 ºC for 4 minutes, followed by 35 cycles of 94 ºC for 45 seconds, 55 ºC for 20 seconds and 66 ºC for 2 minutes. A final extension was performed at 72 ºC for 8 minutes. PCR products were purified after excision from agarose gel, using QIAquick Spin Kits according to the manufacturer’s instructions (Qiagen). Purified PCR-products were transformed into *E. coli* EZ competent cells after ligation into the pDrive cloning vector using the QIAGEN PCR-cloning Kit (Qiagen).

The 16S rDNA fragments were sequenced from the pDrive-clones using the primers M13 reverse: 5′-AACAGCTATGACCATG-3′ and M13f forward: 5′-GTAAAACGACGGCCAGT-3′ described in the Qiagen PCR Cloning Handbook (04/2001). The sequencing was performed using BigDye® Terminator v3.1 Cycle Sequencing Kit (Applied Biosystems). The sequencing program consisted of a initial step at 96 ºC for 1 minute, and 25 cycles of 96 ºC for 30 seconds, 45 ºC (M13r) or 50 ºC (M13f) for 15 seconds and 60 ºC for 4 minutes.

Degenerate primers for amplification of parts of *vioA* and *vioB* genes were designed based on the VioA and VioB amino acid sequences from different violacein producers, retrieved from the GenBank. For amplification of a ~1.0 kb segment encoding the flavoenzyme VioA, the degenerate primer pair VPA3: 5′-CCRCAGCTSCAYCCGCATTTCCAG-3′ and VPA4: 5′-CAGGCYGCCCTCCATCCA GCCRCA-3′ were used. Parts of *vioB*, encoding the heme protein VioB was amplified using two primerpairs. The primerpair VPB1: 5′-CTGTTCAATATGTCGACGCCGC-3′ and VPB2: 5′-GCGGATCGCACATCTGCCACATC-3′ amplificated a ~900 bp strech, and the degenerate primers VPB3: 5′-CCGGCCGGCCGSCTGCTGC-3′, VPB4: 5′-GSCGCGAGCGSCKSAGGCTGC-3′ amplificated a ~1.85 kb segment of *vio*B. The PCR program included initial denaturation at 96 °C for 5 minutes, followed by 35 cycles of 95 °C for 1 minute, 60 °C for 1 minute and 72 °C for 2 minutes. The final extension was performed at 72 °C for 10 minutes. The 1.0 kb segment of *vioA* and the ~900 bp segment of *vioB* were cloned as described for 16S rDNA–sequences. Sequencing was performed by Eurofins MWG Operon.

The phylogenetic analyses of the cloned sequences were performed using MEGA 4 [38]. Sequences were aligned with their closest hits from BLAST searches, trimmed to the same length, and the phylogenetic trees constructed using neighbour-joining with 2000 or 1000 bootstrap replicates. Comparing the sequences with other available 16S rDNA and vioA/B sequences were done by BLAST searches to determine strain homology and identity. DNA sequences reported in this study have been deposited to GenBank under accession numbers GQ160908, GQ160909, GU062792 and GU062793.

## Figures and Tables

**Table 1.** Bacterial strains and plasmids used in this study.

**Strain or plasmid**	**Description**	**Source or reference**
*Candida albicans*	Cyh^r^ AmB^s^, Hp^s^, Nys^s^†	ATCC• (strain 10231)
*Micrococcus luteus*	Amox^s^, Amp^s^, Cm^s^, Ccl^s^, Nb^s^, Ole^s^, Pen^s^, Pcn^s^, Rif^s^, Ty^s^£	ATCC• (strain 9341)
*E. coli* K12		
*Enterococcus faecium*	Amp^r^, Ctc^r^, Ery^r^, Lcm^r^, Vcm^r^, Am^r^, Bac^r^, Cs^r^, Sp^r^§	CCUG* (strain 37832)
*Enterococcus faecium Collimonas* CT	Am^r^, Bac^r^, Cs^r^, Sp^r^	CTC (strain 492) This study

• The American type Culture Collection.

† Cyh: cycloheximide, AmB: amphotericin B, Hp: haloprogin, Nys: nystatin.

£ Amox: amoxicillin, Amp: ampicillin, Cm: clindamycin, Ccl: cyclacillin, Nb: novobiocin, Ole: oleandomycin, Pen: penicillamine, Pcn: penicillin, Rif: rifamycin, Ty: tylosin.

§ Amp: ampicillin, Ctc: chlortetracyclin, Ery: erythromycin, Lcm: lincomycin, Vcm: vancomycin, Am: apramycin, Bac: bacitracin, Cs: cycloserine, Sp: spectinomycin.

* Culture Collection, Gothenburg University.
